# Evaluation of a Mobile Phone Image-Based Dietary Assessment Method in Adults with Type 2 Diabetes

**DOI:** 10.3390/nu7064897

**Published:** 2015-06-17

**Authors:** Megan E. Rollo, Susan Ash, Philippa Lyons-Wall, Anthony W. Russell

**Affiliations:** 1 Priority Research Centre in Physical Activity and Nutrition and School of Health Sciences, University of Newcastle, Callaghan, New South Wales 2308, Australia; 2 School of Exercise and Nutrition Sciences, Faculty of Health, and Institute of Health and Biomedical Innovation, Queensland University of Technology, Kelvin Grove, Queensland 4059, Australia; E-Mail: s.ash@qut.edu.au; 3 School of Exercise and Health Sciences, Edith Cowan University, Joondalup, Western Australia 6027, Australia; E-Mail: p.lyons-wall@ecu.edu.au; 4 Department of Diabetes and Endocrinology, Princess Alexandra Hospital, Woolloongabba, Queensland 4102, Australia; E-Mail: Anthony.Russell2@health.qld.gov.au; 5 School of Medicine, University of Queensland, Woolloongabba, Queensland 4102, Australia

**Keywords:** diabetes, doubly labelled water, image-based dietary records, nutrition assessment

## Abstract

Image-based dietary records have limited evidence evaluating their performance and use among adults with a chronic disease. This study evaluated the performance of a 3-day mobile phone image-based dietary record, the Nutricam Dietary Assessment Method (NuDAM), in adults with type 2 diabetes mellitus (T2DM). Criterion validity was determined by comparing energy intake (EI) with total energy expenditure (TEE) measured by the doubly-labelled water technique. Relative validity was established by comparison to a weighed food record (WFR). Inter-rater reliability was assessed by comparing estimates of intake from three dietitians. Ten adults (6 males, age: 61.2 ± 6.9 years old, BMI: 31.0 ± 4.5 kg/m^2^) participated. Compared to TEE, mean EI (MJ/day) was significantly under-reported using both methods, with a mean ratio of EI:TEE 0.76 ± 0.20 for the NuDAM and 0.76 ± 0.17 for the WFR. Correlations between the NuDAM and WFR were mostly moderate for energy (*r* = 0.57), carbohydrate (g/day) (*r* = 0.63, *p* < 0.05), protein (g/day) (*r* = 0.78, *p* < 0.01) and alcohol (g/day) (*r_s_* = 0.85, *p* < 0.01), with a weaker relationship for fat (g/day) (*r* = 0.24). Agreement between dietitians for nutrient intake for the 3-day NuDAM (Intra-class Correlation Coefficient (ICC) = 0.77–0.99) was lower when compared with the 3-day WFR (ICC = 0.82–0.99). These findings demonstrate the performance and feasibility of the NuDAM to assess energy and macronutrient intake in a small sample. Some modifications to the NuDAM could improve efficiency and an evaluation in a larger group of adults with T2DM is required.

## 1. Introduction

Nutrition therapy provided by a dietitian and self-management education and support are important strategies for the effective long-term management of type 2 diabetes mellitus (T2DM) [1]. The measurement of dietary intake is necessary to inform, support and evaluate these interventions. Traditional prospective methods of recording intake, such as weighed or estimated food records, are ideal as they allow for the natural day-to-day variation in intake to be captured [2], however these methods are often associated with high burden and changes to usual intake [3,4,5].

Image-based dietary records continue to show promise in alleviating the issues associated with subject burden relating to the collection of dietary intake information among adults [6,7], including those with T2DM [8]. Evaluation of the performance of image-based dietary records as an independent prospective method to estimate nutrient intake in adults has predominantly been limited to relative validity [9,10,11,12,13] and inter-rater reliability [11,13,14]. However, evaluation with an objective reference method is essential to determine the true accuracy or criterion validity (defined as the comparison to a criterion value to determine the extent to which the test method captures a true representation of the dietary variable it intends to measure [15]). The doubly labelled water (DLW) technique is a method used to assess total energy expenditure (TEE) and is considered the “gold standard” method to validate self-reported dietary energy intake (EI) [16,17]. Only one study [7] has determined criterion validity of self-reported energy intake (EI) derived from image-based dietary records.

Therefore, this study aimed to establish the preliminary validity (both relative and criterion) and inter-rater reliability of the Nutricam Dietary Assessment Method (NuDAM) in adults with T2DM. The usability and acceptability of the NuDAM in this group was also assessed.

## 2. Experimental Section

### 2.1. Subjects and Study Design

In this pilot study, a pre-determined sample size of 10 adults with T2DM was used with subjects recruited through a research study database and internal university staff email list serves. To be eligible to participate in the study, subjects needed to meet the following criteria: be aged 18–70 years; have a diagnosis of T2DM of >3 months; not currently receiving treatment for cancer or have a previous diagnosis of liver, kidney or thyroid diseases; not currently trying to lose weight; and have a stable body weight (assessed as not having lost or gained more than 4 kg in the past 6 months). The study was approved by the Queensland University of Technology Human Research Ethics Committee and each subject provided written informed consent.

For the evaluation of new dietary assessment methods it is recommended that test and reference methods are used separately, with the test method used first [18]. Therefore, dietary intake was assessed using the NuDAM (test method) in week 1 and the weighed food record (WFR) (reference method) in week 2. Intake was assessed over a three day period (two week days and one weekend day; non-consecutive) for both methods. Demographic information was collected on Day 0, in addition to height to the nearest 0.1 cm using a stadiometer (Model PE087, Mentone Educational, Moorabbin, Australia) and body weight to the nearest 0.1 kg using calibrated electronic scales (Model HD319, Tanita Corporation, Tokyo, Japan). Weight was also measured on Days 8 and 15. To account for factors which may explain mis-reporting of intake [17], dietary restraint was also measured on Day 0 using a 10-item scale [19]. At the end of each dietary recording period, subjects were asked to complete a brief questionnaire on the experience of using the NuDAM and the WFR. Response options to questions included Likert and categorical scales in addition to open-ended text.

### 2.2. Total Energy Expenditure (TEE)

TEE was measured over a two week period using the DLW technique and coincided with the collection of dietary intake using the NuDAM and WFR. Administration of the DLW occurred on Day 0, with subjects in a fasted state. Subjects were orally dosed with 1.25 g 10% ^18^O + 0.1g 99% ^2^H/kg and a post-dose urine sample was collected 6 hours after drinking the DLW. During Days 1–14 subjects were required to collect one urine sample each day. The level of enrichment of ^18^O and ^2^H isotopes contained in the urine samples were measured in triplicate by isotope ratio mass spectrometry (Hydra 20/20 CF-IRMS, Sercon, Cheshire, UK). Isotope dilution spaces were derived [20] and used to calculate carbon dioxide production [21]. Indirect calorimetry principles were applied and TEE derived via using the modified Weir [22] equation, with a standard respiratory quotient of 0.85 used for all subjects.

### 2.3. Nutricam Dietary Assessment Method (NuDAM)

**Figure 1:**
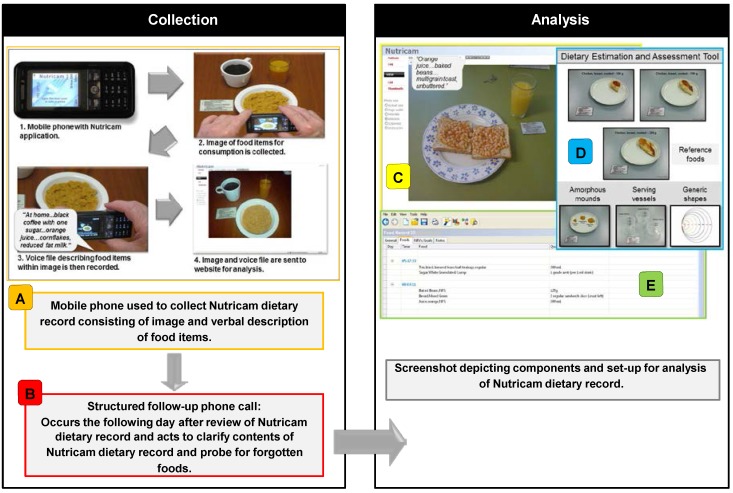
Overview of the Nutricam Dietary Assessment Method (NuDAM). For the collection of dietary intake data, a mobile phone is used to capture the Nutricam image-based dietary record (**A**) and is combined with information collected via a phone call (using a standardized interview protocol) (**B**) Analysis consisted of the dietitian identifying and quantifying food items contained in each Nutricam dietary record entry (**C**) A standardized protocol and the Dietary Estimation and Assessment Tool (DEAT) (a two-dimensional portion size estimation aid (**D**)) was used to assist in the task of quantifying the food items. Dietary data was entered directly into the nutrient analysis software program, FoodWorks^®^ (**E**) to obtain an estimate of nutrient intake. Data from the follow-up phone call (**B**) is used to supplement the Nutricam dietary record, with adjustments made by the dietitian to the analysis (**E**) as required.

The NuDAM consisted of a prospective mobile phone Nutricam image-based dietary record and brief phone call to the subject the following day (Figure 1). Details of the development and early testing of Nutricam have been described previously [8]. Building on this earlier work, the NuDAM method was modified to incorporate a follow-up phone call component to clarify items in the Nutricam record and probe for commonly forgotten foods. In addition, the current study used a standardized analysis protocol including an aid (called the Dietary Estimation and Assessment Tool) to assist in the quantification of food portions contained in the images.

The Nutricam dietary record was recorded using a Sony Ericsson K800i mobile phone (Sony Ericsson Mobile Communications AB, Lund, Sweden) installed with the software application Nutricam (Alive Technologies, Pty. Ltd., Arundel, Australia). When recording the image, subjects were instructed to place the reference object (a 9 cm × 5 cm card which also acted as a prompt for recording an entry) next to the food items, hold the phone at an angle of approximately 45° and ensure all items were clearly visible. After capturing the image, subjects were automatically prompted to make a voice recording describing the location, meal occasion, and the foods (name, type, brand/product name, and preparation/cooking method) contained in the image. Information documenting any food leftover at the end of the eating occasion was also collected in a similar manner. All subjects were trained in the use of the Nutricam mobile phone prior to the collection of the 3-day dietary record and were provided with written instructions for reference during the recording period. The Nutricam record was automatically sent to a secure website accessed only by the researchers. Additional intake information consisting of the clarification of foods within the Nutricam record and probing for forgotten foods was collected from subjects during a brief structured phone call by a Dietitian (D1) on the morning following each recording day.

### 2.4. Weighed Food Record (WFR)

In the second week, dietary information was collected using a 3-day WFR. Subjects were provided with a set of digital food scales (Model HR 2385, Koninklijke Philips Electronics N.V., Amsterdam, The Netherlands) (accurate to 1 g) and were required to weigh all food items prior to consumption and record all information (including recipes) into the paper-based diary supplied. Any food which was served and recorded in the diary but then not eaten was also required to be weighed and documented. At the completion of the recording period, the WFR was reviewed by a dietitian (D1) in the presence of the subject to ensure that the information was complete.

### 2.5. Nutrient Analysis from the NuDAM and WFR

The two sets of dietary records were analysed independently by three dietitians (D1 and two additional dietitians, D2 and D3) using the *AUSNUT 1999* food composition databases [23] in the nutrient analysis software program FoodWorks^®^ Professional 2009 (Xyris Software, Brisbane, Australia). The Nutricam dietary records were analysed first. Using both the image and accompanying voice recording for each eating occasion, each dietitian identified and quantified food items contained in the Nutricam records and entered this information directly into the nutrient analysis program. To assist with the quantification of foods in the images, each dietitian used a portion size estimation aid, called the Dietary Estimation and Assessment Tool (DEAT), previously developed by the research team (Figure 1). The tool consisted of various reference images of foods, serving vessels, amorphous mounds and generic shapes and was based on aids developed for other dietary assessment methods [24,25]. The reference object (9 cm × 5cm card) also appeared in the DEAT and provided perspective to the dietitian during the analysis. Dietitians were then provided with a recording of the phone calls to each subject following each Nutricam recording day and used this information to make any adjustments to the NuDAM analysis. For the WFRs, information on the types and amounts of foods consumed contained within the diaries was entered directly into the FoodWorks^®^ program.

### 2.6. Statistical Analysis

Data analysis was performed using SPSS for Windows (version 17.0, 2008, SPSS Inc., Chicago, IL, USA). For both dietary assessment methods, the estimates of energy and macronutrient intake were averaged for the three days for each subject and then separately for each of the three dietitians. Intra-class correlation coefficients (ICC) evaluated agreement between dietitians’ estimates of energy and macronutrient intake for each method. Repeated-measures ANOVA or Friedman’s ANOVA were used to assess differences between dietitians’ estimates (Bonferroni correction post hoc analysis applied). Paired *t*-tests or Wilcoxon signed-rank test assessed differences in the overall nutrient intake (average of the three dietitians’ estimates) between methods and for EI and TEE. Correlation coefficients were used to determine the relationship between estimates of nutrient intake derived from the NuDAM and WFR. Validation of self-reported EI was based on the principle of EI = TEE ± body stores, where in the absence of non-significant weight change at the group level, the expected ratio of EI:TEE is 1.00 [17]. At the individual level with the 95% confidence limits (CL) calculated to determine mis-reporting [26]. The calculated 95% CL for the NuDAM were 0.72 and 1.28; and for the WFR were 0.76 and 1.24.

## 3. Results

Six men and four women with T2DM ranging in age between 48–69 years participated, with all 10 subjects completing the study. Five were classified as obese (body mass index (BMI) ≥30.0 kg/m^2^), four as overweight (BMI 25.0–29.9 kg/m^2^), and one was within the normal BMI range (18.5–24.9 kg/m^2^). The group showed a low level of dietary restraint, with individual scores ranging between 1.3 to 3.2 (out of 5). At the group level, there were no significant changes in mean body weight during Week 1 (baseline to Day 8), −0.7 ± 1.2 kg, Week 2 (Day 8 to Day 15), 0.4 ± 0.9 kg and overall (baseline to Day 15) −0.3 ± 1.2 kg.

### 3.1. Criterion and Relative Validity

The overall mean EI was 8.8 ± 2.0 MJ/day from the NuDAM and 8.8 ± 1.8 MJ/day from the WFR; both were significantly lower than mean TEE of 11.8 ± 2.3MJ/day (*p* < 0.01). The mean EI:TEE ratio was 0.76 ± 0.20 and 0.76 ± 0.17 for the NuDAM and WFR, respectively. At the individual level, three males and four males were classified as under-reporters for the NuDAM and WFR, respectively. NuDAM under-reporters were also found to be under-reporting EI with the WFR. When using the NuDAM, all three under-reporters of EI lost weight in the first recording week (−2.8 kg each for two subjects and −0.3 kg for one subject). In comparison among the under-reporters identified using the WFR, two subjects had no change in weight while the other two subjects gained weight (+0.6 kg and +2.4 kg) in the second week. No individuals were found to be over-reporting EI. Overall, the mean nutrient intakes were not significantly different between the two dietary assessment methods (Table 1). Associations between intakes were stronger for protein and alcohol, moderate for energy and carbohydrate, and weaker for fat.

### 3.2. Inter-Rater Reliability

The inter-rater reliability and comparison of the dietitians’ estimated energy and nutrient intakes from the NuDAM and WFR are shown in Table 1 Bonferonni post-hoc analysis between dietitians showed estimates by D1 to be significantly different for energy compared to both D2 and D3, protein compared to D3, and fat and carbohydrate compared to D2.

### 3.3. Usability, Acceptability and Changes to Eating Behaviours

All subjects preferred to use the NuDAM to record intake compared to the WFR, with “convenience”, “ease of use”, and “portability” used to explain preferences. All subjects would be willing to use both recording methods again. For the Nutricam mobile phone, the majority (*n* = 9) would be willing to use again to record their intake for periods of up 7 days or longer, whereas up to 3 days was the maximum recording period most commonly reported (*n* = 5) for the WFR. Subject responses to additional questions relating to the experience of the NuDAM and WFR are summarized in Table 2.

**Table 1:** Comparison of energy and nutrient intake obtained from NuDAM and WFR between dietitians and between methods (*n* = 10 subjects).

		Mean(± SD) Intake as Assessed by Each Dietitian ^†^			ICC (95% CI) between Dietitians	Overall ^‡^	
		D1	D2	D3		Mean ( ± SD) Intake ^§^	Correlation ^ between Methods
Energy (MJ/day)	NuDAM	8.2 ± 1.7	9.0 ± 2.3 *	9.1 ± 2.0 *	0.88 (0.58–0.98) ***	8.8 ± 2.0	0.57
	WFR	8.5 ± 1.6	8.9 ± 2.0	8.9 ± 1.8	0.92 (0.80–0.98) ***	8.8 ± 1.8	
Protein (g/day)	NuDAM	89.3 ± 20.2	99.0 ± 31.4	98.1 ± 23.1 *	0.79 (0.53–0.94) ***	95.5 ± 23.7	0.78 **
	WFR	89.1 ± 26.8	91.9 ± 28.2	91.5 ± 24.9	0.97 (0.92–0.99) ***	90.8 ± 26.4	
Fat (g/day)	NuDAM	75.6 ± 18.3	87.0 ± 25.4 *	86.6 ± 20.1	0.77 (0.45–0.93) ***	83.1 ± 20.3	0.24
	WFR	79.5 ± 16.8	85.4 ± 27.4	80.9 ± 24.2	0.82 (0.59–0.95) ***	81.9 ± 21.8	
CHO (g/day)	NuDAM	194.9 ± 52.8	212.0 ± 52.7 *	215.3 ± 60.8	0.91 (0.71–0.98) ***	207.4 ± 54.4	0.63 *
	WFR	206.3 ± 53.8	207.2 ± 54.9	211.9 ± 57.8	0.92 (0.79–0.98) ***	208.5 ± 53.9	
Alcohol (g/day) ^#,¶^	NuDAM	15.0 ± 29.4	13.6 ± 28.0	14.4 ± 29.5	0.99 (0.98–0.99) ***	14.3 ± 28.9	0.85 **
	WFR	16.1 ± 23.4	17.4 ± 30.2	16.5 ± 28.4	0.99 (0.98–0.99) ***	16.7 ± 28.3	

Abbreviations: D1: dietitian No.1; D2: dietitian No.2; D3: dietitian No.3; CHO: carbohydrate; NuDAM: Nutricam dietary assessment method; WFR: weighed food record; ^†^ Repeated-measures ANOVA (GLM) between dietitians for each dietary method, except for alcohol (^#^) which was Friedman’s ANOVA: * *p* < 0.05, compared to D1, all others not significant; ICC: Intra-class Correlation Coefficient significant: *** *p* < 0.001; Difference within each dietitian’s mean estimates of nutrient intake, NuDAM *vs.* WFR (paired *t*-test or ^#^ Wilcoxon Signed Ranked test): not significant; ^‡^ Overall mean (± SD) intake = mean (D1, D2, and D3 intake per day); ^§^ difference between overall mean (± SD) estimate of nutrient intake, NuDAM *vs.* WFR: not significant for energy or macronutrient intakes; ^ Correlations are Pearson’s correlation coefficient (*r*); except for alcohol (^¶^) which is Spearman’s rank correlation coefficient (*r_s_*): * *p* < 0.05, ** *p* < 0.01.

**Table 2:** Evaluation of Nutricam dietary assessment method and weighed food record (*n* = 10 subjects).

Questions (as Presented):	Count			
Usability and Acceptability ^	Strongly Agree	Agree	Neutral	Disagree
Overall, I found the Nutricam mobile phone easy to use:	7	2	1	0
Overall, I found weighing my foods and drinks easy:	0	3	4	3
NuDAM only:				
I found taking photographs of food and drink items easy *:	5	5	0	0
I found recording the voice file easy *:	5	5	0	0
I found that the Prompt Card was useful for remembering how to use Nutricam:	5	1	4	0
When prompted during the call: I found it easy to clarify the details of the food and/or drink items that I had eaten during the previous day:	8	1	1	0
I found it easy to remember if there were any food and/or drink items I had not recorded using the Nutricam mobile phone the previous day:	7	3	0	0
I found it easy to remember the description of the food and/or drink items I had not recorded using the Nutricam mobile phone the previous day:	6	4	0	0
I found it easy to remember the quantities of the food and/or drink items I had not recorded using the Nutricam mobile phone the previous day:	6	3	1	0
Overall, I found that the length of the calls I received were appropriate:	5	4	1	0
Change to eating behaviours	No		Yes	
Was there any difference in how you used the Nutricam mobile phone to record your diet when you were alone compared to when you were with other people or in public?	4		6	
Was there any difference in how you recorded your diet using the weighed record method when you were alone, compared to when you were with other people or in public?	2		8	
Did you record all food and drink items that you consumed during the test period using the Nutricam mobile phone?	5		5	
Did you record all food and drink items that you consumed during the test period using the weighed record method?	4		6	
Where there any foods and/or drinks that you usually eat, but did not eat during the Nutricam test period?	9		1	
Where there any foods and/or drinks that you usually eat, but did not eat during the weighed record method test period?	6		4	

Abbreviations: ^ These questions were answered on a 5-point Likert Scale (Strongly agree/Agree/Neutral/Disagree /Strongly disagree); however no responses for the “strongly disagree” category were recorded; * Questions refer to using the Nutricam mobile phone to collect the image-based dietary record.

Changes in eating behaviours were reported for both methods (Table 2). More than half of the subjects reported a difference in how the methods were used when in the presence of others as opposed to when they were alone. The most common reason for this response was feeling more self-conscious and/or requiring to explain why they were recording their intake when in public compared to at home. Regardless of the method used, forgetting to record prior to eating was the main reason for not recording all food items consumed. Making changes to the types of foods typically consumed was more common for the WFR, with simplifying intake in order to facilitate recording often reported for this method.

## 4. Discussion

This study assessed the criterion and relative validity and the inter-rater reliability of the NuDAM for the estimation nutrient intake, with the findings demonstrating the performance and feasibility of this method in a small sample of adults with T2DM. Compared to TEE, similar levels of under-reporting of EI were found for the NuDAM (−23.7%) and WFR (−23.9%). The level of under-reporting for the NuDAM is comparable to using 3-day food records where the difference between EI and TEE may be up to −24% in older adults [27,28,29]; and more favourable to using a 3-day food recall in obese adults with T2DM where a difference of up −60% was reported [30]. Martin *et al.* [7] used DLW to validate EI collected over 6 days using a mobile phone image-based dietary record among free-living overweight and obese adults. When used with generic meal time reminders sent to the phone mean participant error between EI and TEE was −34.3%, compared to when the reminders were tailored to the specific meal times of the individual under-reporting decreased to −3.7% [7]. The combination of a longer recording period and customised meal-time prompts may have contributed to the greater reporting accuracy and will be considered for future use of the NuDAM.

The associations between the NuDAM and WFR for estimated intakes of energy, protein, and carbohydrate were similar to some studies [9,10], although others have found stronger correlations [11,12]. Compared to these studies, estimates of fat intake between the NuDAM and WFR showed a weaker relationship (*r* = 0.24). However in these studies intake was recorded concurrently and therefore differs from our study where records were collected one week apart and higher within-subject variation is expected. Alcohol intake was highly correlated between methods (*r_s_* = 0.85) and displayed the strongest agreement between dietitians. The use of standardized serving vessels and detailed descriptions (e.g., “pint” glass) may have contributed to the strength of the relationship observed for alcohol. It is important to note, the observed correlations for estimates of energy and nutrient intake between the two dietary assessment methods are based on the assumption that the errors between the methods are independent [18]. Therefore the validity of the NuDAM should be interpreted in the context of the other measures of agreement.

Inter-rater reliability for the nutrients assessed ranged from moderate to high for the NuDAM. Although discrepancies existed between dietitians’ nutrient estimates for the NuDAM, these did not translate to significant differences between methods in the overall mean intakes of the group. Similar studies have also found acceptable agreement between dietitians for estimates of nutrient intake derived from image-based records [13,14]. While these studies were conducted in controlled settings of single meal occasions or using pre-prepared food items, the NuDAM was used in a free-living situation over multiple days with opportunity for greater food variety.

Nine subjects were classified as either overweight or obese in our study and this may in part explain the level of reporting accuracy observed. Increasing BMI is generally associated with an increase in the likelihood of under-reporting, however some variation does occur at the individual level [17]. The dietary restraint scale used measured both actual restriction of intake and intention to restrict [31], with scores of ≤3 categorised as “low-restraint” [32]. In the current group, overall dietary restraint was low, even among those subjects identified as under-reporting intake who all had restraint scores ≤3 Therefore, the level of dietary restraint did not appear to influence the accuracy of self-reported EI in the current study.

Forgetting to record intake prior to consumption was commonly reported as reasons for not collecting all intake information for both the NuDAM and WFR and remains a challenge with prospective dietary records. Although, the phone call component of the NuDAM was designed to capture food items consumed but not recorded, it is possible that selective mis-reporting may have been present. Snacks and foods eaten at times other than main meals are most commonly mis-reported [33] and could also explain the difference between TEE and EI observed in our study.

Change in behaviours were reported for both methods, although there appeared to be a slightly greater change in eating behaviours during the period recording with the WFR compared to the NuDAM. At the individual level, those identified as under-reporters of EI using the NuDAM all lost weight and may suggest under-eating [34]. In contrast with the WFR, two of the four under-reporters had no change in weight which may be indicative of under-recording of intake [34]. However, replication in a larger sample would be necessary to confirm these conclusions regarding changes in intake and/or recording using the NuDAM and WFR. An increased awareness and changes to intake behaviours are common when diet is recorded [3,4,5], including when wearable devices are used to automatically collect image-based records [35]. Further exploration into the effect of using wearable devices and mobile/smartphone to collect image-based records has on eating behaviours and dietary intake is needed.

Similar to other studies which have found a preference for image-based methods over traditional dietary assessment methods [8,9,10,11], the NuDAM was also well received among this group of older adults with T2DM. Subjects were willing to use the NuDAM again and for longer recording periods. However, some refinement to the method could be incorporated to improve efficiency in the collection of the dietary data, such as replacing the follow-up phone call with an in-built feature in the Nutricam application to collect missed eating occasions. As use of the method in its current form may not be feasible in large groups, further modifications to the NuDAM are required to minimize the effect on analysis time that occurred with shifting some of the subject burden to the dietitian. New techniques which automate all or some of the quantification of foods within the image-based dietary record hold promise for improving efficiency in the analysis [36,37].

Strengths of this study are the use of a “gold standard” DLW technique to validate EI and the use of standardized analysis protocol, including aids to estimate portion size of foods in the Nutricam records. Limitations include the small sample which restricts generalisability of these results to the greater population of adults with T2DM. However, when using DLW, small samples have been used initially to validate measures of EI [17] and justify evaluation in larger numbers. It is possible that the use of the NuDAM first may have introduced a training effect for the WFR. The administration sequence of the two dietary assessment methods was standardised for all subjects and based on recommendations [18], however randomisation of the administration order will be considered for future NuDAM validation studies. The use of the same dietitian (D1) (MER) to review and clarify the dietary data and then to code the records is another potential limitation. Although, a standardized protocol was followed for all dietitians, increased familiarity with the subject intakes in the NuDAM and WRF could have contributed to the difference in nutrient intake estimates.

## 5. Conclusions

This pilot study assessed the validity (criterion and relative) and inter-rater reliability of a novel image-based dietary assessment method, the NuDAM, in 10 adults with T2DM. The results demonstrated that in comparison to an objective measure of TEE the NuDAM performed equally well to the WFR, however EI was significantly under-estimated by both methods. Relative validity was comparable to other image-based prospective food records for all nutrients, except for fat. Agreement between dietitians for estimates of nutrient intake was slightly lower for the NuDAM compared to WFR. All subjects preferred using the NuDAM and were willing to use it again for longer recording periods. These findings demonstrate the performance and feasibility of the NuDAM to assess energy and macronutrient intake in a small group of adults with T2DM. However, some modifications to the method are necessary to improve efficiency, particularly for use with a greater number of individuals. Evaluation in a larger group is needed to be able to generalise the results to the broader population of adults with T2DM.
